# Clinical Outcome Comparison of Patients Requiring Extracorporeal Membrane Oxygenation With or Without COVID-19 Infection

**DOI:** 10.7759/cureus.39078

**Published:** 2023-05-16

**Authors:** Flora S Park, Aalap C Shah, Sonali Rao, Joseph Rinehart, Kei Togashi

**Affiliations:** 1 Anesthesiology and Perioperative Medicine, University of California Irvine Health, Orange, USA

**Keywords:** ventilator, va-ecmo, vv ecmo, venovenous extracorporeal membrane oxygenation, venoarterial extracorporeal membrane oxygenation, outcomes, comparison, respiratory failure, extracorporeal membrane oxygenation, covid-19

## Abstract

In severe COVID-19-related respiratory failure, extracorporeal membrane oxygenation (ECMO) is a useful modality that is used to provide effective oxygenation and ventilation to the patient. This descriptive study aimed to investigate and compare the outcomes between COVID-19-infected patients and patients who were not infected and required ECMO support. A retrospective study was undertaken on a cohort of 82 adult patients ($\geq$18-year-old) who required venoarterial (VA-ECMO) and venovenous (VV-ECMO) ECMO between January 2019 and December 2022 in a single academic center. Patients who were cannulated for COVID-19-related respiratory failure (C-group) were compared to patients who were cannulated for non-COVID etiologies (non-group). Patients were excluded if data were missing regarding cannulation, decannulation, presenting diagnosis, and survival status. Categorical data were reported as counts and percentages, and continuous data were reported as means with 95% confidence intervals. Out of the 82 included ECMO patients, 33 (40.2%) were cannulated for COVID-related reasons, and 49 (59.8%) were cannulated for reasons other than COVID-19 infection. Compared to the non-group, the C-group had a higher in-hospital (75.8% vs. 55.1%) and overall mortality rate (78.8% vs. 61.2%). The C-group also had an average hospital length of stay (LOS) of 46.6 ± 13.2 days and an average intensive care unit (ICU) LOS of 44.1 ± 13.3 days. The non-group had an average hospital LOS of 24.8 ± 6.6 days and an average ICU LOS of 20.8 ± 5.9 days. Subgroup analysis of patients only treated with VV-ECMO yielded a greater in-hospital mortality rate for the C-group compared to the non-group (75.0% vs. 42.1%). COVID-19-infected patients may experience different morbidity and mortality rates as well as clinical presentations compared to non-COVID-infected patients when requiring ECMO support.

## Introduction

The ongoing SARS-CoV-2 pandemic continues to put pressure on healthcare systems around the world with the resurgence of resistant strains. Severe organ dysfunction from COVID-19 infection is often debilitating or fatal and is associated with respiratory failure and multi-organ system failure [1]. In cases of severe COVID-19-related respiratory failure, extracorporeal membrane oxygenation (ECMO) is a useful and effective treatment modality that is often used as a last resort [2, 3]. ECMO can be venovenous (VV-ECMO) or venoarterial (VA-ECMO). To treat respiratory distress syndrome (ARDS), which often develops in severe cases of COVID-19, VV-ECMO is mainly used in COVID-19 patients experiencing respiratory failure [4]. During VV-ECMO, blood is removed from the venous system, passed through a membrane oxygenator by a centrifugal pump, and returned to the venous system through inflow and outflow cannulas [3]. This (VV-ECMO) effectively provides oxygenation and ventilation to the patient who suffers from respiratory failure. While ECMO can aid in the treatment of patients with COVID-19 infection and respiratory failure, the impact of ECMO on COVID-19 outcomes has not been well documented.

Our study aimed to describe the clinical outcomes of COVID-19-infected patients receiving ECMO management and to elucidate how these outcomes differed from those of patients with non-COVID-19 infection causes.

## Materials and methods

A retrospective cohort study was performed on adult ($\geq$18 years old) patients who required ECMO between January 2019 and December 2022 in a single academic center. The definition of acute respiratory distress syndrome (ARDS) was based on the Berlin definition [5]. The decision to provide ECMO management was based on: 1) patients who have received conventional respiratory support for less than two weeks (invasive or non-invasive ventilator management); 2) developing life-threatening respiratory acidosis (pH $&lt;$7.3 and hypoxia PaO2/FiO2$&lt;$100); 3) BMI $\leq$45; and 4) patients who do not have any other organ failure. Patients who were cannulated for COVID-19-related respiratory failure (C-group) were compared to patients who were cannulated for non-COVID etiologies (Non-group).

Demographic data included age, sex, body mass index (BMI), American Society of Anesthesiologists (ASA) physical classification score, the reason for cannulation, total hospital length of stay (LOS), and intensive care unit (ICU) LOS. The type of ECMO that patients received was also noted (VV-ECMO or VA-ECMO). Patients with missing information were excluded. Clinical outcome data collected included in-hospital mortality and overall mortality. A follow-up of patient status was conducted via chart review on December 28, 2022. Patients with missing variables were excluded from the analysis.

Descriptive statistics were used to characterize the study population. Categorical data were reported as counts and percentages. Continuous data were reported as means with a 95% confidence interval (CI). All analyses were performed using IBM Statistical Package for Social Sciences (SPSS) Statistics for Macintosh (Version 28, IBM Corp., Armonk, NY). A subgroup analysis was performed for patients with and without COVID-19 who received VV-ECMO.

## Results

A total of 82 patients required ECMO management during the study period. No patients were excluded. The non-group's average age was 54 ± 4.2 years, while the C-group's average age was 47 ± 3.8 years. All patients presented with PaO2/FiO2 $&lt;$100, and a PaCO2 increase ($>$45mmHg) resulting in respiratory acidosis with arterial blood gas pH $&lt;$ 7.35 at the time of ECMO consideration. The most common diagnostic reason for ECMO cannulation in the C-group was pneumonia, while the most common reasons for cannulation in the non-group were cardiovascular etiologies (Table 1).

**Table 1:** Demographics of non-COVID and COVID patients receiving ECMO Categorical and continuous outcomes are tabulated as n (%) and mean ± 95% confidence interval, respectively. VV-ECMO: venous extracorporeal membrane oxygenation; ECMO: extracorporeal membrane oxygenation; BMI: body mass index; ARDS: acute respiratory distress syndrome; 1: unlisted etiologies included blunt trauma, burns, and autoimmune disease

Characteristic	Non-COVID ECMO patients (n=49)	COVID ECMO patients (n=33)
Age, years	54 ± 4.2	47 ± 3.8
Male sex	30 (61.2%)	26 (78.8%)
VV-ECMO	19 (38.8%)	32 (97.0%)
BMI, kg/m^2^	29.3 ± 2.0	37.9 ± 4.2
Indications for ECMO		
Cardiovascular	32	0
ARDS	6	1
Asthma	1	0
Pneumonia	2	32
Pulmonary embolism	2	0
Pleural empyema	1	0
Other^1^	5	0

Of the total cohort, 30 patients (36.6%) survived transfer or discharge from the hospital. There were 33 patients who were cannulated for COVID-19 infection-related etiology, of whom eight patients (24.2%) survived to either hospital transfer or discharge. Seven of the eight patients (21.2%) survived until follow-up. Of the 49 patients who were cannulated for non-COVID-19 infection etiologies, 22 patients (44.9%) survived to either hospital transfer or discharge, 19 (38.8%) of whom survived until the last follow-up. The length of time on the ventilator for the non-group was 17.0 ± 5.3 days, and the time on the ventilator for the C-group was 36.7 ± 10.1 days. The time until cannulation for the non-group was 7.6 ± 3.7 days, while for the C-group it was 1.2 ± 1.1 days. The non-group had an in-hospital mortality rate of 55.1% and an overall mortality rate of 61.2%, while the C-group had an in-hospital mortality rate of 75.8% and an overall mortality rate of 78.8%. Hospital and ICU LOS for the non-group were 24.8 ± 6.6 days and 20.8 ± 5.9 days, respectively, while for the C-group these durations were 46.6 ± 13.2 days and 44.1 ± 13.3 days (Table 2).

**Table 2:** Clinical outcomes of non-COVID and COVID patients receiving ECMO Categorical and continuous outcomes are tabulated as n (%) and mean ± 95% confidence interval, respectively. ECMO: extracorporeal membrane oxygenation; AKI: acute kidney injury; LOS: length of stay; ICU: intensive care unit

Characteristic	Non-COVID ECMO patients (n=49)	COVID ECMO patients (n=33)
ECMO duration, days	10.8 ± 3.8	35.5 ± 10.2
AKI	34 (69.4%)	18 (51.5%)
Stroke	13 (26.5%)	9 (27.3%)
Hospital LOS, days	24.8 ± 6.6	46.6 ± 13.2
ICU LOS, days	20.8 ± 5.9	44.1 ± 13.3
Ventilator days	17.0 ± 5.3	36.7 ± 10.1
Time until cannulation, days	7.6 ± 3.7	1.2 ± 1.1
In-hospital mortality	27 (55.1%)	25 (75.8%)
Overall mortality	30 (61.2%)	26 (78.8%)

A greater proportion of the C-group received VV-ECMO compared to the non-group (97.0% vs. 38.8%) (Table 1) and required a longer duration of ECMO support (35.5 ± 10.2 days vs. 10.8 ± 3.8 days) (Table 2). One patient in the C-group started with VV-ECMO cannulation for three days, switched to VA-ECMO for two days, and then switched back to VV-ECMO for the remainder of the ECMO treatment.

VV-ECMO patients were found to experience longer ECMO duration (29.5 ± 7.3 days vs. 6.1 ± 3.8 days), hospital LOS (43.3 ± 9.5 days vs. 17.5 ± 7.1 days), and ICU LOS (39.5 ± 9.2 days vs. 14.7 ± 6.9 days) regardless of ECMO cannulation etiology (Table 3). When the VV-ECMO subgroup of patients was examined, the mean ECMO duration in COVID-19 patients was 34.6 ± 10.4 days. Hospital LOS and ICU LOS in this cohort were 44.9 ± 13.3 days and 42.3 ± 13.2 days, respectively, and the in-hospital mortality rate was 75.0% while the overall mortality rate was 78.1% (Table 4).

**Table 3:** Clinical outcomes of VA-ECMO patients compared to VV-ECMO patients Categorical and continuous outcomes are tabulated as n (%) and mean ± 95% confidence interval, respectively. VA-ECMO: venoarterial extracorporeal membrane oxygenation; VV-ECMO: venovenous extracorporeal membrane oxygenation; ECMO: extracorporeal membrane oxygenation; AKI: acute kidney injury; LOS: length of stay; ICU: intensive care unit

Characteristic	VA-ECMO patients (n=31)	VV-ECMO patients (n=51)
ECMO duration, days	6.1 ± 3.8	29.5 ± 7.3
AKI	17 (54.8%)	35 (68.6%)
Stroke	4 (12.9%)	18 (35.3%)
Hospital LOS, days	17.5 ± 7.1	43.3 ± 9.5
ICU LOS, days	14.7 ± 6.9	39.5 ± 9.2
Ventilator days	17.2 ± 7.4	29.6 ± 7.4
Time until cannulation, days	5.2 ± 2.2	12.8 ± 3.5
In-hospital mortality	20 (64.5%)	32 (62.7%)
Overall mortality	21 (67.7%)	25 (68.6%)

**Table 4:** Clinical outcomes of non-COVID and COVID patients receiving VV-ECMO Categorical and continuous outcomes are tabulated as n (%) and mean ± 95% confidence interval, respectively. VV-ECMO: venovenous extracorporeal membrane oxygenation; ECMO: extracorporeal membrane oxygenation; AKI: acute kidney injury; LOS: length of stay; ICU: intensive care unit

Characteristic	Non-COVID ECMO patients (n=19)	COVID ECMO patients (n=32)
ECMO duration, days	21.3 ± 7.7	34.6 ± 10.4
AKI	18 (94.7%)	17 (53.1%)
Stroke	9 (47.4%)	9 (28.1%)
Hospital LOS, days	40.6 ± 12.4	44.9 ± 13.3
ICU LOS, days	34.8 ± 11.0	42.3 ± 13.2
Ventilator days	19.6 ± 8.8	35.6 ± 10.2
Time until cannulation, days	11.1 ± 8.7	1.2 ± 1.1
In-hospital mortality	8 (42.1%)	24 (75.0%)
Overall mortality	10 (52.6%)	25 (78.1%)

## Discussion

We present one of the few studies describing clinical outcomes in patients requiring ECMO support for COVID-19 infection and non-COVID-19 infection-related etiologies spanning the duration of the pandemic. Previous studies report an overall in-house mortality rate of around 40% for COVID-19 infection-related patients requiring VV-ECMO [6-9]. Additionally, mortality rates vary for different COVID-19 strains, with those that arose later in the pandemic (i.e., Delta) having the highest mortality rate with ECMO [8]. In our study, all COVID-19-infected patients but one received VV-ECMO and underwent longer ECMO duration, both in the ICU and in the hospital, compared to non-COVID-19-infected patients.

Our in-hospital (75.8%) and overall mortality rates (78.8%) for patients infected with COVID-19 were higher than previous reports [6-8]. This difference could be due to the wide time span for data collection, which included COVID-19 patients presenting at different time points of the pandemic. Also, there was a wide variation in treatment protocols for COVID-19 all across the referral centers. Additionally, differences in resource allocation criteria may have affected clinical outcomes in patients managed on ECMO. Especially at our high-volume medical center, ECMO support was provided when conventional ARDS management failed, including ventilator support. This difference in ECMO management allocation criteria may have contributed to the differences in patient acuity and the eventual requirement for treatment length.

Of particular note was our finding that ECMO patients infected with COVID-19 demonstrated high hospital and ICU LOS. During the COVID-19 pandemic, ICUs were operating at near capacity, potentially resulting in an increased length of time required for patients to get transferred to a secure ICU bed and also an increased length of time between ICU admission and VV-ECMO cannulation due to resource scarcity. This could potentially lead to an increased duration of mechanical ventilation prior to ECMO cannulation [10]. However, this finding is in contrast to other studies, which report no significant difference in in-hospital or long-term mortality in COVID-19-infected patients compared to patients without COVID-19 [11, 12].

Previous findings have reported a different etiology of respiratory distress in COVID-19 patients compared to those presenting with pneumonia and ARDS, leading to prolonged ECMO support and higher mortality [9, 13]. A continuous risk-benefit evaluation of ECMO therapy is necessary. Although definitions of futility are institution-specific, some centers consider returning to conventional management if no lung or cardiac recovery is noted after approximately 21 days [14]. At our center, goals of care conversations were held with the family when no clinical improvement was observed 14-21 days after initiating ECMO support.

Early clinical decompensation of COVID-19 may be associated with improved outcomes compared with late decompensation. There are studies documenting increased survival rates when early VV-ECMO is initiated [15, 16]. Unfortunately, we did not see a difference in survival due to the timing of ECMO initiation.

Kurihara et al. noted that while there were no significant differences in mortality, patients infected with COVID-19 and receiving ECMO had a greater rate of thrombotic and bleeding complications than non-COVID-19 ECMO patients [12, 17]. Additionally, Zheng et al. reported more severe pneumonia and cardiovascular complications in COVID-19-infected patients with underlying cardiovascular disease [18]. In addition to pre-existing co-morbidities leading to more severe disease pathology, patients infected with COVID-19 could have been more likely to receive ECMO and experience its complications. In a previous study by Tan et al., longer ECMO duration was significantly associated with higher incidences of bloodstream infections (BSI) and ventilator-associated pneumonia (VAP) [19]. Although, at first glance, in the VV-ECMO subgroup, we found what seems to be a lower rate of AKI and stroke in COVID-19 patients, these are only two complications studied without any comparative analysis, so further studies must be conducted to confirm these findings.

There were several limitations to our study. In addition to the inherent limitations of a single-center retrospective study of a hospital database, the results may not be representative of outcomes at other institutions due to differences in resource availability and allocation criteria. A multi-center study with a larger sample size is warranted to yield more clinically significant findings and increase generalizability for patient outcomes. Our study was designed to be strictly descriptive, so no hypothesis testing was performed. Due to a lack of robust clinical parameter collection, we were not able to draw any conclusions on the impacts of specific co-morbidities on clinical outcomes, including the high mortality rate for COVID-19 patients requiring ECMO support. Data regarding specific complications during ECMO cannulation was also not collected, so conclusions regarding the complication rate and its association with mortality were not able to be drawn. Also, while the Survival after Veno-Arterial ECMO (SAVE) and Respiratory Extracorporeal Membrane Oxygenation Survival Prediction (RESP) scores are effective predictors of outcomes for VA-ECMO and VV-ECMO patients, respectively, these scores were not collected in this study and should be examined further in future studies [20, 21].

## Conclusions

This study provided a descriptive analysis of clinical outcomes in a single-institution cohort of COVID-19-infected and non-COVID-19 patients receiving ECMO support. Patients suffering from COVID-19 infection receiving ECMO had higher hospital and ICU LOS and a tendency for higher mortality compared to patients with non-COVID-19 infection etiology. Due to the lasting effects of the COVID-19 pandemic, it is crucial to illuminate the existing differences in outcomes arising from COVID-19 complications requiring ECMO support. Due to limitations regarding study design, further studies investigating the differences in outcomes noted in this study are warranted.
